# The effect of intraocular pressure during phacoemulsification in patients with either diabetic retinopathy or glaucoma; a randomized controlled feasibility trial

**DOI:** 10.1007/s00417-025-06839-0

**Published:** 2025-04-29

**Authors:** Raffaele Raimondi, Karmen Sow, Tunde Peto, Nicholas Wride, Maged S. Habib, Alan Sproule, Alyson K. Muldrew, Michael Quinn, David H. Steel

**Affiliations:** 1 Sunderland Eye Infirmary, Sunderland, UK; 2 Centre for Public Health, Queen’s University of Belfast, Belfast, UK; 3 Bioscience Institute, Newcastle University, Newcastle Upon Tyne, UK

**Keywords:** Phacoemulsification, Intraocular pressure, Diabetic retinopathy, Glaucoma, Complications, Surgical efficiency

## Abstract

**Purpose:**

To investigate whether performing phacoemulsification with a lower infusion pressure using the Centurion active sentry system affects surgical efficiency, complications and a range of clinical and imaging parameters compared to the higher pressures routinely used in patients with cataract and concomitant diabetic retinopathy and glaucoma.

**Setting:**

Sunderland Eye Infirmary, Sunderland, United Kingdom.

**Design:**

Masked observer randomized controlled feasibility trial.

**Methods:**

Patients with cataracts undergoing routine phacoemulsification with either diabetic retinopathy or primary open-angle glaucoma of any severity were included and randomized to an infusion pressure of 30 (‘LOW’) or 60 (‘HIGH’) mmHg. All other fluidic settings were standardized. Surgical metrics and a range of imaging and clinical variables were measured pre- and postoperatively on days 1, 21 and 40.

**Results:**

Seventy eyes from 70 patients underwent surgery and completed follow-up. Forty-one patients had diabetic retinopathy and 29 had glaucoma. There was no difference in any of the recorded surgical metrics including cumulative dissipated energy (CDE) between the two randomization groups (mean CDE 6.5 versus 6.1 percent seconds in the HIGH and LOW groups respectively, p = 0.68). There were no patients in either group with posterior capsule rupture or other intraoperative complications.

There was no significant difference in the number of patients with raised intraocular pressure (IOP) on day 1. Seven (21.2%) patients in the LOW and 5 (13.3%) in the HIGH group had slit lamp detectable corneal oedema on day 1, which had all resolved by day 21. There were no between group differences for visual acuity, IOP, corneal thickness, and any of the optical coherence tomography (OCT) acquired measures at any of the time points.

The foveal avascular zone perimeter and area were significantly smaller on day 21 than at baseline in the HIGH group as compared to the LOW group (P = 0.03 and 0.04 respectively), with a corresponding increase in the superficial vascular plexus density (p = 0.04).

**Conclusion:**

Using an infusion pressure of 30mmHg with standardized aspiration fluidic settings on the Centurion active sentry system did not decrease surgical efficiency or increase complication rates compared to a pressure of 60mmHg. The lower infusion pressure may cause fewer short-term changes in the retinal microvasculature, the long-term significance of which is unknown.

**Key messages:**

***What is known***

- Traditionally, phacoemulsification has been carried out under relatively high intraocular pressure (IOP) to mitigate the effects of post occlusion aspiration surge during lens removal.
-  A new enhanced phacoemulsification fluidics system has reduced surge allowing surgeons to operate at considerably lower, and more physiological IOP levels.

***What is new***

- In patients undergoing phacoemulsification for moderate cataracts with either co-existing diabetic retinopathy or glaucoma, an IOP of 30 mmHg using the Centurion active sentry system did not result in any decrease in surgical efficiency or increase in complication rates compared to a higher pressure of 60 mmHg.
- Lower IOP phacoemulsification caused less short-term changes in the retinal microvasculature than higher pressure, the long-term significance of which is unknown and further study is needed.

**Supplementary Information:**

The online version contains supplementary material available at 10.1007/s00417-025-06839-0.

## Introduction

Since theintroduction of phacoemulsification, several technological improvements have been made to improve the safety of surgery and machine efficiency. One advance has been in the reduction in post occlusion break or surge. This occurs if the phacoemulsification tip becomes fully occluded by lens material with secondary vacuum build up to the preset maximum. When occlusion is broken, fluid rushes from the anterior chamber (AC) towards the machine cassette, lowering intraocular pressure (IOP), shallowing the AC and risking posterior capsule rupture [1].Traditionally, the infusion pressure has been raised to partly reduce the effect of surge and these have had to be raised further with time due to reductions in incision size [2].

There have been concerns that operating with these supranormal pressures could be detrimental. Intraocular pressure changes have been noted to alter retinal blood flow after glaucoma drainage procedures, but the effect of phacoemulsification alone has not been studied [3, 4]. There have however been reports of glaucoma progression following cataract surgery in the past and it was postulated that these could be related to high IOP or fluctuations in IOP [5, 6]. Recent studies have supported these hypotheses with reductions in choroidal and central retinal artery blood flow during raised IOP [7–9]. The association between non-arteritic anterior ischemic optic neuropathy and cataract surgery has also been documented [7]. Indeed a variety of short- and long-term changes in both retinal structure and the retinal vasculature have been described after phacoemulsification [8–10].

An enhanced fluidics system that actively manages irrigation combined with a handpiece that incorporates an IOP pressure sensor allowing rapid fluidic responses (Centurion Vision System combined with Active Sentry Handpiece, Alcon Laboratories, USA) has considerably reduced surge. This has enabled surgeons to operate with higher vacuum levels improving power use efficiency. It has also permitted surgeons to operate at considerably lower IOP levels than previously, in the physiological range [11–14].

It is not known if low IOP settings improve the outcomes of surgery, as compared to higher IOP settings, particularly in patients with diabetic retinopathy or glaucoma, where the effects of the strategy are likely to be of greatest benefit. There are however also some evidence that operating with raised IOP may have advantages in terms of surgical efficiency with reduced energy use, chatter and increased aspiration rates reported in laboratory studies [15, 16].

We hypothesized that lower infusion pressure combined with standardized aspiration fluidic settings using the Centurion active sentry system would not result in reduced surgical efficiency nor an increased rate of complications as compared to the higher pressures widely used currently. Furthermore, we hypothesized that a low IOP might result in a lower prevalence of induced alterations in the retinal vasculature, or neural retina in patients with diabetic retinopathy and glaucoma. The aim of this study was therefore to assess these parameters in a randomized feasibility trial of patients with either glaucoma or diabetic retinopathy undergoing phacoemulsification, to provide initial findings and guide future trials.

## Methods

### Study design

This was a masked observer randomized controlled feasibility trial of a low (30 mmHg) infusion pressure (referred hereon as the ‘LOW’ group) as compared to a standard pressure (60 mmHg) (‘HIGH’ group) during phacoemulsification. The protocol was agreed in advance and the trial was registered on clinicaltrials.gov (NCT04637685). Surgeries were performed by 3 experienced phacoemulsification surgeons in a tertiary ophthalmology center in the United Kingdom (UK); Sunderland Eye Infirmary, Sunderland. All surgeons had performed over 5000 cataract surgeries prior to study start. UK multicenter ethical approval was obtained (reference 21/LO/0067, IRAS 288743). After a comprehensive discussion, all patients signed a written consent form.

### Recruitment criteria

We included patients with cataract undergoing routine phacoemulsification with either diabetic retinopathy or primary open angle glaucoma of any degree of severity.

We excluded patients with dense cataracts (greater than Lens Opacities Classification System, version II categories N3, C2, P2) [17] precluding adequate retinal imaging, posterior polar cataracts, subluxated cataract, the presence of other vision affecting pathology, uncontrolled intraocular inflammation, myopia > − 6 diopters or axial length > 26 mm, amblyopia, and any conditions affecting the ability to perform imaging including head tremor and positioning problems. We also excluded patients who had had laser, intravitreal anti vascular endothelial growth factor injections or steroids either at the time of surgery or in the 6-months prior to surgery. Only one eye per patient was recruited.

Preoperative visual acuity, axial length, intraocular pressure, pupil size, and diabetic retinopathy grade were recorded.

### Participants, randomization and masking

Randomization to the study intervention of a low infusion pressure was carried out by research staff using online randomization, with a block size of 2 (REDCap, https://projectredcap.org/about/) immediately prior to surgery, with stratification by surgeon and disease type.

Patients were masked to randomization group. All outcome assessors (research nurses, medical assessor, vision testing team, and photographers) were masked to treatment group allocation. Optical coherence tomography (OCT) and OCT angiograms (OCTA) were graded by an accredited reading center (NetwORC UK, Central Administrative Resource Facility, Queen’s University Belfast, Northern Ireland, UK) with images being transferred with anonymized study number only attached. The surgeon carrying out the surgery was not masked to the randomization group.

### Surgical procedure

Other than the infusion pressure, all other phacoemulsification settings were standardized, with 2.2 mm clear corneal temporal incisions and a 1 mm side port. Management of small pupil was standardized across arms with the use of pupil rings if needed. An Active Sentry hand piece with balanced tips was used in both groups. Pure linear torsional phacoemulsification with ‘Intelligent Phaco’ set on and set to deliver 5 milli-second bursts of longitudinal power when the vacuum reached 95% of the commanded level was set. A ‘stop and chop’ surgical technique was used by all 3 surgeons. Vacuum settings, phacoemulsification power settings along with aspiration flow settings were standardized between the two groups. (Supplementary Table 1).

Cumulative dissipated energy (CDE, the average phacoemulsification power multiplied by total phacoemulsification time), total phacoemulsification on-time, volume of infusion solution aspirated and used, longitudinal power-on time and total operation time were recorded. Following surgery topical chloramphenicol was used for 3 days and 1.0% prednisolone acetate and 0.5% ketorolac used for 1 month post-operatively.

## Outcomes:

The study was designed as a feasibility study to assess adverse events and the sample size for definitive follow-on studies and as such all endpoints were exploratory. These included several surgical parameters (CDE, phacoemulsification on-time, volume of infusion solution, longitudinal power-on time and total operation time) as well as best corrected visual acuity (VA), and intraocular pressure at all 3 time points postoperatively.

A range of imaging based exploratory endpoints were also assessed postoperatively including:

- Changes in central subfield thickness (CST) and macular volume.
- Changes in peripapillary retinal nerve fibre layer (RNFL) thickness at a diameter of 3.5 mm.
- Changes in central corneal thickness and anterior chamber depth.
- Changes in foveal avascular zone (FAZ) area and perimeter.
- Changes in central superficial retinal capillary density.
- Changes in sub foveal choroidal thickness.
- Changes in retinal hyper-reflective foci.

### Patients’ ophthalmic examination

A complete ophthalmic examination, including best corrected visual acuity (BCVA) using an ETDRS vision chart at 4 m and slit-lamp biomicroscopy (Haag Streit, Switzerland) was performed preoperatively (baseline) (within 14 days of surgery), and on days 1 and 21 postoperatively. A protocol refraction with assessment of VA was performed on day 40 postoperatively. Complications and adverse events were recorded. The 21- and 40-day time points were chosen based on routine practice at the hospital.

### Imaging protocol and analysis

CST and macular volume were measured at all visits using the Heidelberg Spectralis spectral-domain OCT (Heidelberg Engineering, Heidelberg, Germany) at baseline, days 1, 21 and 40. The scan protocol was a 20⁰ × 20⁰ field, with 120-micron scan spacing and high-speed settings. Perpendicular choroidal thickness was measured sub foveally at all time points on the central scan. The presence of hyper reflective foci was also recorded on the central scan.

**Fig. 1:**
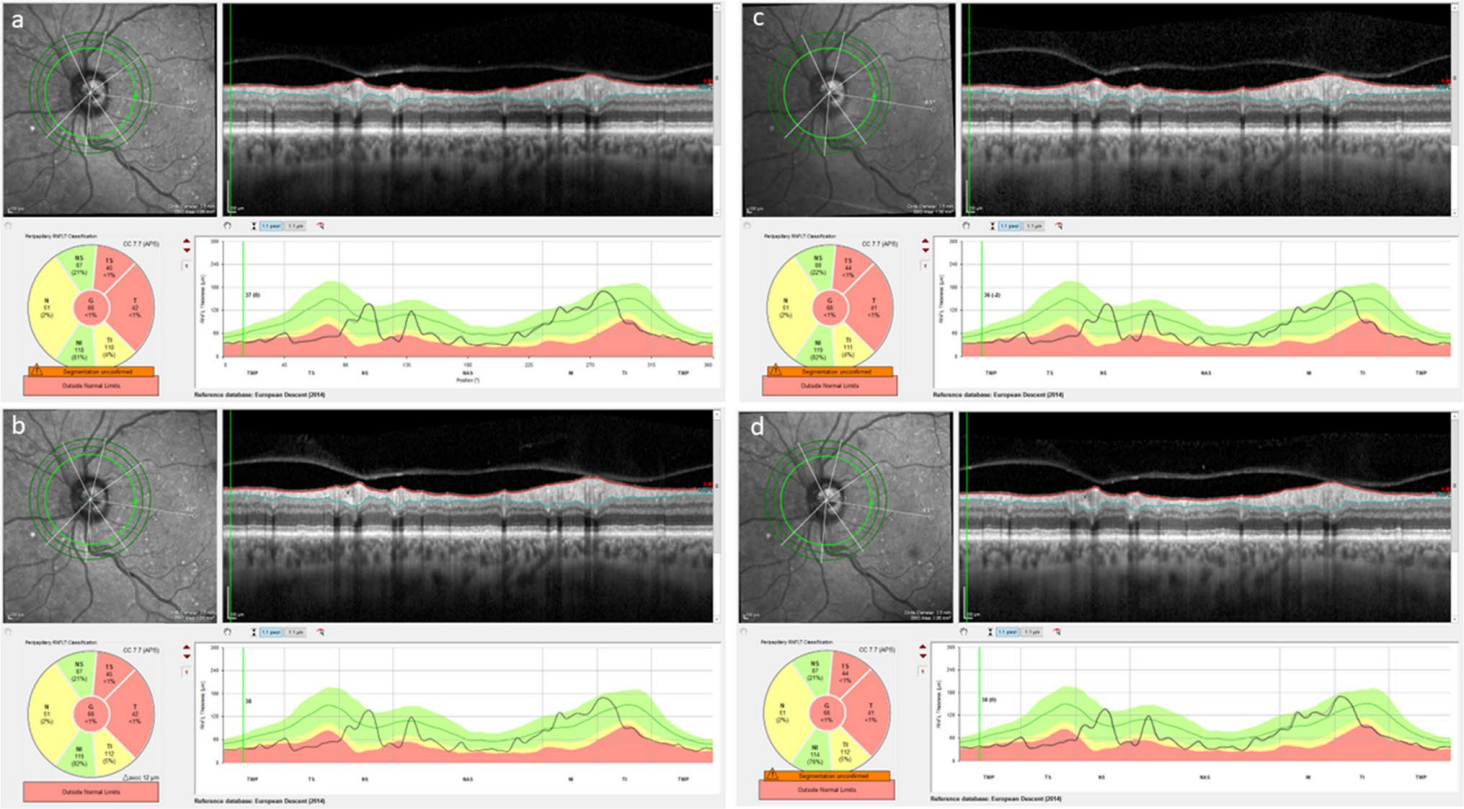
Example of a patient with glaucoma with the optic nerve head-radial and circle scan used at baseline (**a**), day1 (**b**), 21(**c**) and day 40 (**d**) to measure the peripapillary retinal nerve fiber layer at a diameter of 3.5 mm, centered on the Bruch’s Membrane Opening

The Spectralis glaucoma module premium edition (GMPE) optic nerve head-radial and circle (ONH-RC) scan was used at baseline, day 1, 21 and day 40 to measure the peripapillary RNFL at a diameter of 3.5 millimeters (mm), centered on the Bruch’s Membrane Opening (BMO). (Fig. 1) The Heidelberg Anatomic Positioning System was used to ensure that OCT images were acquired at fixed and known retinal locations relative to the center of the fovea and the center of the BMO. All subsequent GMPE scans were aligned to the baseline landmarks and automatically oriented according to the patient’s fovea-BMO center axis. TruTrack eye-tracking was also used to account for changes in head position and ensured precise placement of follow up scans. The global value for the RNFL at a diameter of 3.5 mm was used for analysis.

**Fig. 2:**
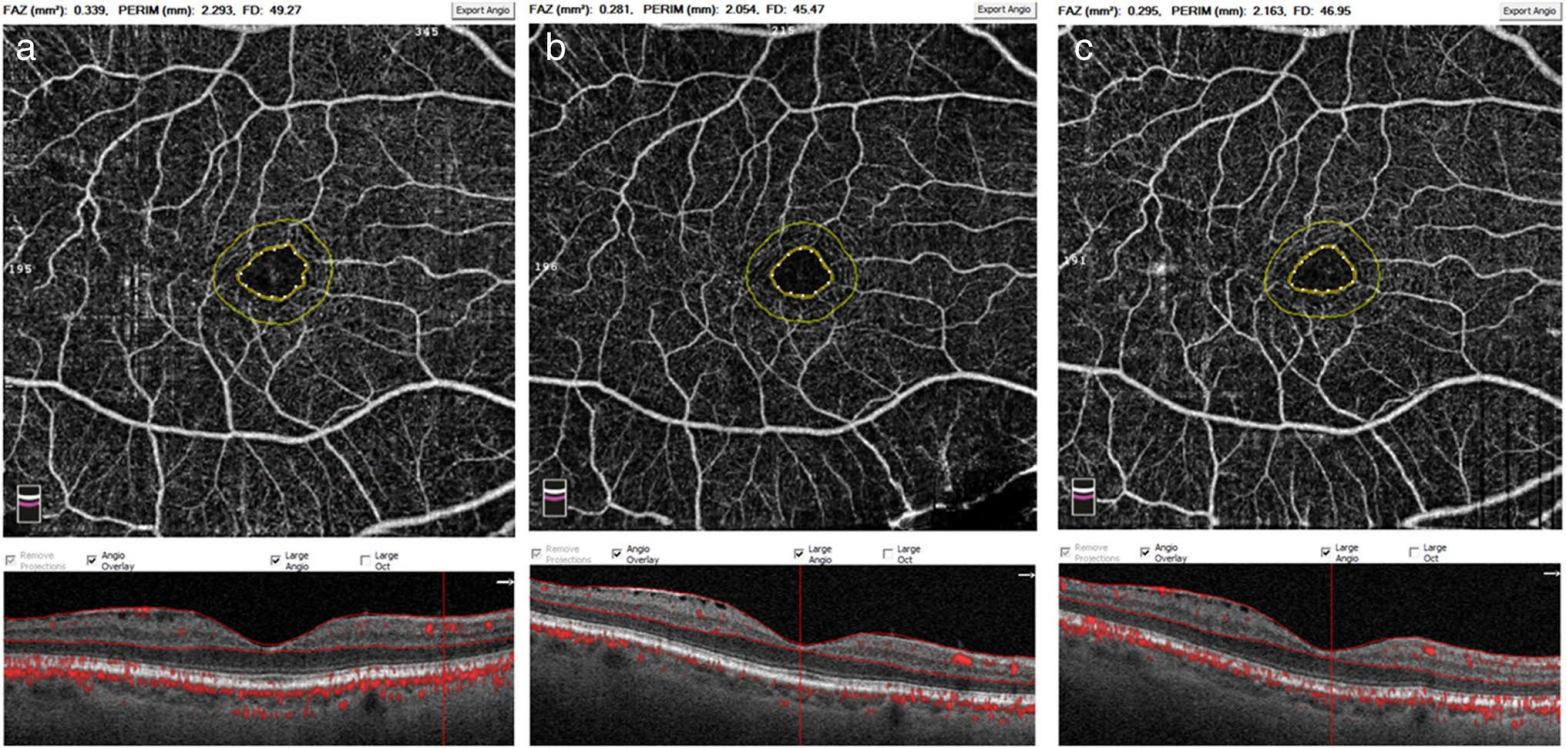
Example of optical coherence tomography angiography performed in a patient with glaucoma and a mild epiretinal membrane at baseline (**a**), day 21 (**b**) and day 40 (**c**) with foveal avascular zone area and perimeter shown

OCTA was performed using RTvue-XR Avanti OCT (Optovue RTVue XR Avanti; Optovue, Inc., Fremont, CA, USA) at baseline, day 21 and day 40). All scans were performed by one experienced technician to ensure the consistency and accuracy of OCTA data. OCTA images of the superficial retinal layers were used to determine the superficial capillary plexus density using the manufacturers AngioVue software. (Fig. 2) The superficial retina was defined as 3 µm below the internal limiting membrane to 15 µm below the inner plexiform layer. The area and perimeter of the FAZ were recorded.

All automatic segmentations were manually checked and adjusted in case of gross segmentation errors by masked graders.

Central corneal thickness and anterior chamber depth were measured using the OCULUS PENTACAM® AXL WAVE (OCULUS INC., Arlington, WA) at all visits.

### Sample size

The study was designed as a feasibility study to assess a range of exploratory endpoints to use for sample size calculations for future studies. We did however use a sample size which would allow us to assess equivalence of CDE used in the two arms. Based on data from our previous study [14] we chose a sample size of 68, which allowed a 90% power to detect a difference of 2 percent seconds in CDE with a standard deviation (SD) of 2.5 and a significance level of 5%. Similarly, the same sample size also allowed us to have a 90% power to assess equivalence in volume of infusion fluid used of 3cc. To allow for drops out we aimed to recruit 72 patients.

In patients for whom both eyes were eligible for the study, the first eye to undergo surgery was included.

### Statistical analysis

An intention-o-treat analysis was carried out. The baseline and demographic characteristics between the two groups were compared. Continuous variables were compared between groups using the unpaired t-test if found to be normally distributed, and the Mann–Whitney test if not normally distributed. Categorical variables were compared between groups using the Chi-square test, except where numbers were small when Fisher’s exact test was performed.

The paired t-test was used to examine changes from baseline within each group. Group differences were analyzed using Analysis of Covariance. The measurement at days 1, 21, and 40 was used as the outcome for each analysis, with the equivalent baseline measurement used as covariate in the analysis. In all tables group differences are expressed as outcome for the LOW group minus the outcome for the HIGH group.

The McNemar test was used to examine changes over time within each group. The Chi-square test was used to compare measurements at days 1, 21 and 40 between groups. Due to the correlation between baseline and follow-up measurements, the baseline values were not adjusted for in the analysis of this outcome.

Analyses were performed for all patients, and then separately for the patients with glaucoma and diabetic retinopathy.

## Results

**Fig. 3:**
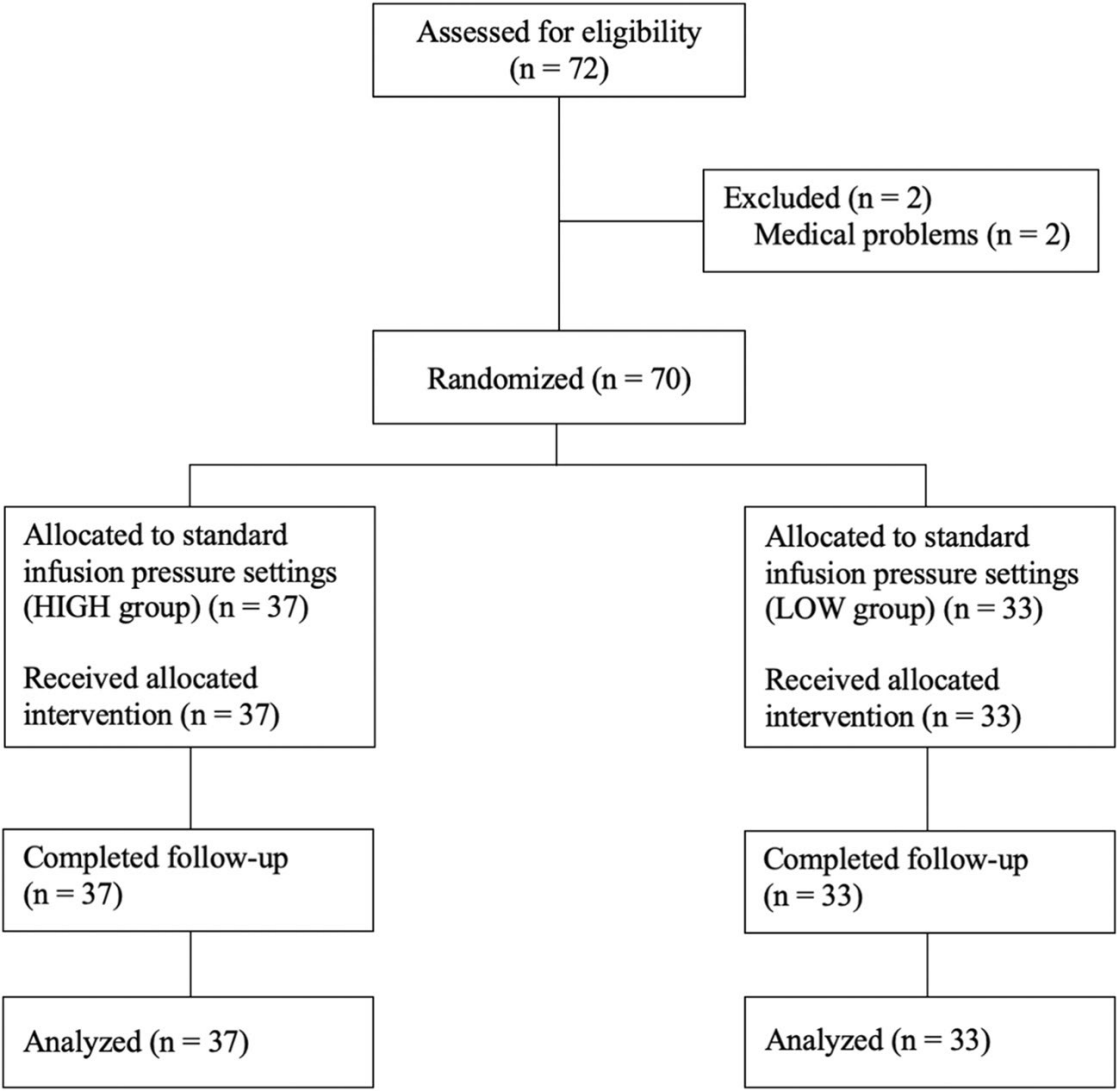
Participant flow diagram of patients who were randomly allocated to HIGH and LOW groups, received intended treatment and were analyzed for the outcomes

Seventy-two eyes of 72 patients were recruited. Two were randomized (one in each randomization group) but were cancelled on the day of surgery due to medical problems, (uncontrolled hypertension and upper respiratory tract infection). All 70 patients who underwent surgery, had the randomized settings used and completed follow up. (Fig. 3).

## Baseline variables

The baseline characteristics of the 70 analyzed patients are shown in Table 1. The baseline variables were well matched between the groups.

**Table 1:** Baseline characteristics

Characteristic	HIGH [N = 37]	LOW [N = 33]	P-value
Age (years)	72.2 ± 9.4 {52, 89}	70.3 ± 8.7 {52, 91}	0.38
Male sex	16 (43%)	18 (55%)	0.35
Right laterality	25 (68%)	16 (48%)	0.11
Baseline best corrected visual acuity (ETDRS letters)	63 [52, 70] {0, 79}	59 [53, 70] {3, 80}	0.91
Glaucoma Diabetic retinopathy	16 (43%) 21 (57%)	13 (39%) 20 (61%)	0.74 0.69
Intraocular pressure (mmHg)	15.1 ± 3.2 {10, 21}	16.5 ± 3.5 {12, 24}	0.07
Axial length (mm)	23.8 ± 1.5 {20.1, 25.9}	23.4 ± 1.5 {20, 25.4}	0.27
Central corneal thickness (microns)	540 ± 36 {461, 623}	541 ± 42 {466, 632}	0.92
Anterior chamber depth (mm)	3.1 ± 0.4 {2.1, 3.8}	3.0 ± 0.5 {2.2, 4.3}	0.35
Sub foveal choroidal thickness (microns)	188 ± 58	213 ± 55	0.08
Hyper-reflective foci present	15 (42%)	9 (32%)	0.44
Pupil size – Small (< 4 mm) – Medium (4 - 6 mm) – Large (> 6 mm)	6 (17%) 19 (53%) 11 (31%)	6 (18%) 17 (52%) 10 (30%)	0.99
LOCS 2 cataract grading -Nuclear sclerosis -Cortical -Posterior subcapsular	2(0–2) {0, 3} 0(0–1) {0, 3} 0(0) {0, 2}	2(0–2) {0, 3} 0(0–1) {0,2} 0(0) {0,3}	0.56

Abbreviations: mm, millimeters; mmHg, millimeters of mercury; LOCS, Lens Opacities Classification System; ETDRS, Early treatment diabetic retinopathy study. Summary statistics are number (percentage), mean ± standard deviation (SD) {range}, or median [inter-quartile range] {range}.

There were 41 patients with diabetic retinopathy recruited and 29 with glaucoma. The baseline characteristics of these subgroups are given in Supplementary Tables 2 and 3. None of the baseline factors varied between randomization groups in either of these subgroups.

## Safety

There were no patients in either group with posterior capsule rupture or other intraoperative complications.

There was no significant difference in the number of patients with raised IOP on day 1. Nine patients (27.3%) in the LOW and 8 (21.6%) in the HIGH group had a day 1 IOP greater than 25.

This represented 2 of the 13 (15.4%) patients with glaucoma in the LOW group and 4 of the 17 (23.5%) patients in the HIGH group.

Similarly, 7 (21.2%) patients in the LOW and 5 (13.3%) in the HIGH group had slit lamp detectable corneal oedema on day 1, which had all resolved by day 21.

There were 6 patients in both groups who had a 10-letters or more drop in visual acuity on day 1 as compared to baseline. All had visual gain compared to baseline on day 40.

## Surgical outcomes

There was no difference in CDE between the two randomization groups (mean CDE 6.5 (SD 3.6) versus 6.1 (SD 3.4) percent seconds in the HIGH and LOW group respectively, p = 0.68). Similarly, there were no significant differences in the other surgical parameters evaluated. There was a non-significant trend towards a shorter case time in the LOW group. (Table 2).

**Table 2:** Surgical outcomes

Characteristic	HIGH [N = 37]	LOW [N = 33]	P-value
Cumulative dissipated energy (percent seconds)	6.5 ± 3.6 {1.5, 18.3}	6.1 ± 3.4 {1.2, 16.9}	0.68
Total ultrasound on time (seconds)	35 [23, 45] {10, 127}	35 [23, 48] {9, 119}	0.97
Volume aspirated (cubic centimeters)	48 [40, 57] {26, 100}	46 [40, 53] {26, 157}	0.46
Longitudinal power on time (seconds)	0.2 [0.1, 0.5] {0.0, 1.4}	0.3 [0.1, 0.6] {0.0, 1.1}	0.48
Total case time (minutes)	7.5 [6.2, 9.2] {4.2, 22.6}	7.0 [6.1, 7.3] {4.1, 22.0}	0.07
Fluid leakage (cubic centimeters)	5.3 [2.1, 6.9] {1.2, 10.8}	4.7 [2.0, 6.2] {1.5, 9.9}	0.49

Summary statistics are number (percentage), mean ± standard deviation {range}, or median [inter-quartile range] {range}.

The analysis results for variables measured over time are summarized in Table 3. There were within group changes over time for several outcomes.

**Table 3:** All variables assessed

Outcome	Timepoint	HIGH group (n = 37)		LOW group (n = 33)		Difference between group
		Mean ± SD	P-value	Mean ± SD	P-value	P-value
BCVA, ETDRS letters	Baseline Day 1 Day 21 Day 40	57 ± 20 61 ± 19 71 ± 15 75 ± 15	0.24 ** < 0.001** ** < 0.001**	56 ± 21 59 ± 21 71 ± 15 75 ± 11	0.45 ** < 0.001** ** < 0.001**	0.84 0.78 0.88
Intraocular pressure, mmHg	Baseline Day 1 Day 21 Day 40	15.1 ± 3.2 20.0 ± 8.7 15.8 ± 4.1 14.3 ± 3.5	** < 0.001** 0.24 0.19	16.5 ± 3.5 21.4 ± 8.0 15.6 ± 3.8 14.8 ± 3.5	**0.001** 0.15 **0.008**	0.91 0.29 0.87
Central corneal thickness, microns	Baseline Day 1 Day 21 Day 40	540 ± 36 639 ± 133 548 ± 40 544 ± 44	** < 0.001** **0.02** 0.16	540 ± 42 665 ± 128 547 ± 48 544 ± 41	** < 0.001** **0.01** 0.15	0.30 0.92 0.94
Anterior chamber depth, millimeters	Baseline Day 1 Day 21 Day 40	3.1 ± 0.4 4.6 ± 0.8 5.2 ± 0.5 5.2 ± 0.5	** < 0.001** ** < 0.001** ** < 0.001**	3.0 ± 0.5 4.4 ± 0.8 5.0 ± 0.4 5.1 ± 0.6	** < 0.001** ** < 0.001** ** < 0.001**	0.34 0.12 0.37
Mean CST, Microns	Baseline Day 1 Day 21 Day 40	301 ± 129 296 ± 127 282 ± 43 288 ± 52	0.85 0.37 0.51	316 ± 180 294 ± 132 280 ± 39 286 ± 48	0.31 0.23 0.34	0.78 0.67 0.77
Macular Volume, cubic millimeters	Baseline Day 1 Day 21 Day 40	8.2 ± 0.8 8.2 ± 0.9 8.4 ± 0.9 8.5 ± 1.0	0.53 **0.002** **0.001**	8.3 ± 0.8 8.2 ± 0.7 8.4 ± 0.7 8.5 ± 0.8	0.27 0.55 0.10	0.56 0.64 0.90
Global peripapillary retinal nerve fibre layer thickness, microns	Baseline Day 1 Day 21 Day 40	81 ± 18 83 ± 16 84 ± 19 85 ± 19	**0.01** ** < 0.001** ** < 0.001**	79 ± 17 83 ± 16 84 ± 19 86 ± 17	**0.006** ** < 0.001** ** < 0.001**	0.24 0.41 0.14
Sub foveal choroidal thickness, microns	Baseline Day 1 Day 21 Day 40	188 ± 58 184 ± 60 199 ± 60 198 ± 58	0.05 0.18 **0.02**	213 ± 55 198 ± 65 210 ± 59 217 ± 60	0.05 0.31 0.06	0.54 0.75 0.84
Retinal hyper-reflective foci, number	Baseline Day 1 Day 21 Day 40	15 (42%) 13 (42%) 15 (42%) 18 (49%)	0.32 1.00 0.32	9 (32%) 11 (38%) 12 (40%) 13 (40%)	1.00 0.56 0.56	0.75 0.89 0.44

Macular volume significantly increased at days 21 and 40 compared to baseline in the HIGH group, although no equivalent significant differences were observed in the LOW group. Global RNFL increased significantly from baseline over time in both groups. Central corneal thickness increased significantly at day 1 in both groups, with large increases of 100-microns compared to baseline values but returning to baseline values by day 40. IOP values were significantly higher at day 1 than baseline in both groups. In the LOW group, IOP values were significantly lower than at baseline at day 40, by a mean of 1.8 mm mercury (mmHg). No equivalent significant difference was observed in the HIGH group. Sub foveal choroidal thickness decreased near significantly in both groups at day 1 and then increased in both groups to day 40, significantly in the HIGH group.

None of these measures however were significantly different between the groups.

In terms of OCTA measures (see Table 4) both FAZ perimeter and FAZ area were significantly lower at day 21 than at baseline in the HIGH group, but there was no difference between these timepoints in the LOW group. Notably, both FAZ perimeter and FAZ area were significantly different between the groups at Day 21.

**Table 4:** Optical coherence tomography angiography findings

Outcome	Timepoint	HIGH group		LOW group		Difference between group
		Mean ± SD	P	Mean ± SD	P	P
Foveal avascular zone perimeter length, millimeters	Baseline	2.20 ± 0.67		2.12 ± 0.52		
	Day 21	2.11 ± 0.60	**0.02**	2.15 ± 0.48	0.36	**0.03**
	Day 40	2.16 ± 0.73	0.50	2.16 ± 0.55	0.92	0.57
Foveal avascular zone area, squared millimeters	Baseline	0.29 ± 0.13		0.29 ± 0.12		
	Day 21	0.28 ± 0.13	**0.03**	0.29 ± 0.12	0.37	**0.04**
	Day 40	0.28 ± 0.15	0.40	0.30 ± 0.13	0.79	0.67
Superficial vascular plexus density	Baseline	39.4 ± 4.2		40.1 ± 5.9		
	Day 21	42.6 ± 4.6	** < 0.001**	40.4 ± 5.8	0.54	**0.04**
	Day 40	42.4 ± 5.8	**0.002**	41.0 ± 5.8	0.15	0.28

Abbreviations; mmHg, millimeters of mercury; IOP, intraocular pressure; CST, central subfield thickness; BCVA, best corrected visual acuity; ETDRS, early treatment diabetic retinopathy study; SD, standard deviation.

Measurements at baseline and day 40 were not significantly difference for both FAZ perimeter and area in both groups. Consistent with these changes the superficial vascular plexus measurements were significantly denser in the HIGH group at day 21, but not day 40.

The analysis results for variables measured over time are summarized in Supplementary Table 4 for glaucoma patients, and Supplementary Table 5 for diabetic retinopathy patients. The results largely mirrored the results for the merged patients group. The day 1 IOP increase was significant in the HIGH group in the glaucoma patients but not in the LOW group, although there was not a significant difference between the groups.

There was a significant difference in FAZ area and perimeter between the HIGH and LOW groups at day 21 in the glaucoma patients as in the whole patient group. Interestingly there were no significant differences in these parameters in the diabetic retinopathy patients.

## Discussion

### Main findings

The main finding of this study is that operating at a more physiological IOP (30 mmHg) is as efficient in terms of energy usage and volume of infusion used as traditionally raised IOP settings (60 mmHg). Similarly, there was no difference in complication rates between the two IOP settings. The two groups had similar outcomes for visual acuity, corneal clarity, IOP, central retinal thickness and RNFL changes. Importantly however we also found that surgery with a lower IOP in eyes with glaucoma induces less short-term changes in the retinal microvasculature than a higher pressure, with changes in the foveal capillaries in the higher-pressure group, not seen in the low IOP group.

### Surgical efficiency

Two previous randomized controlled trials (RCTs) comparing high and low IOP during cataract surgery with gravity fed infusion systems have adjusted the surgical parameters with lower aspiration rates and vacuums in the lower IOP groups to avoid the risks of surge. The lower aspiration settings in the lower IOP groups led to longer case time, and higher infusion volumes as well as CDE [18, 19]. Higher vacuum settings are known to increase surgical efficiency and decrease infusion volume confounding the interpretation of the infusion pressure randomisation.

There have been three other very recent RCTs comparing low and higher IOPs during surgery with the Centurion and Active Sentry systems with fixed aspiration settings. Two of the studies reported no differences in CDE, case time and ultrasound time like the current study [15, 16]. Low IOP settings with matching of all other surgical parameters did not appear to have an adverse effect on surgical efficiency. It can be hypothesized that as the fluidic technology ensured a more constant IOP, CDE was mostly influenced by the aspiration settings which were fixed between the two groups. Another recent paired eye RCT of 27 patients however found a borderline significant reduction in CDE and a reduced infusate volume in the low IOP group [20]. The increase in infusion volume in this study (mean 40 versus 56 ml (ml)) can be partly explained by an 8 ml difference in fluid leakage which we did not observe. We found a lower level of leak at approximately 5 mls in both groups. It is possible that the higher leak levels Rauen et al. observed in their high IOP group also effected their CDE differences.

Interestingly, we did find a trend to a shorter case time in the LOW IOP group with a mean time of 7 min compared to 7.5 in the HIGH group (P = 0.07). In a recent retrospective study of 208 eyes that had undergone surgery with IOP settings of 20 mmHg versus 60 mmHg with otherwise matched fluidic settings, there was a significant reduction in surgical time with lower number of surge events and active sentry activations in the 20 mmHg group, perhaps accounting for the difference [21].

### Safety

High IOP and flow rates have previously been associated with early postoperative corneal oedema [19, 22, 23]. We did not find any difference in this and several other safety outcomes assessed with no difference in visual acuity, IOP, or corneal thickness on day 1 between the groups. Rauen et al. in their RCT reported a lower increase in central corneal thickness and clarity at day 1 in their lower IOP group and a lower decrease in endothelial cell density after surgery. As already mentioned they observed a lower infusate volume in their low IOP group which we did not, which might explain the difference. [20]

### Microvascular changes

We found a significant reduction in FAZ area and perimeter in the HIGH group at day 21 that was not observed in the LOW group. Several previous prospective observational studies have reported a reduction in FAZ area following phacoemulsification [24–26].These studies have also reported a corresponding increase in central macular thickness postoperatively and both finding have persisted until last follow up at 3 months. Several theories have been proposed for the paradoxical reduction in FAZ and increase in vascular density with phacoemulsification. These include the release of cytokines causing vasodilation, the effect of increased light exposure with corresponding increased metabolic activity in the retina, the effect of increased retinal perfusion with the reduction in IOP observed after cataract surgery and finally the effect of increased vessel density because of cleared media. Rauen et al. also found a significant reduction in FAZ area 7 days postoperatively in their higher-pressure group as compared to the lower IOP group, who did not have any significant change FAZ area. The FAZ area returned to baseline levels by day 28 similar to our findings. The same RCT also reported less anterior segment inflammation as assessed by the presence of flare and cells in the anterior chamber on day 1 in the low IOP group, supporting the hypothesis that IOP related cytokine release may have been responsible. [20]

To go along with the reduced FAZ we found an increase in vascular density in the HIGH group. A recent retrospective observational study of two infusion pressures during surgery noted similar changes in vascular density and also that ocular blood flow in the ophthalmic, central retinal, and posterior ciliary arteries were less affected at lower IOP levels [27]. Interestingly the FAZ and vascular density differences that we found were only observed in the eyes with glaucoma and not in the diabetic group, perhaps relating to the loss of autoregulation with diabetic retinopathy [28].There have been reports of FAZ size and vessel density being associated with both the severity of glaucoma and its progression [29, 30]. The finding that low infusion pressure during surgery affects the retinal vasculature less in patients with glaucoma may therefore have particular long term significance. Further long term studies with appropriate sample sizes are needed however to provide specific guidance.

### Retinal thickness

High infusion pressure can also affect retinal thickness. In a prospective series of 30 healthy eyes undergoing phacoemulsification, a greater amount of time at high IOP was associated with increased macular thickness postoperatively [31]. Nourinia et al. also noted that the extent central macular thickening after phacoemulsification positively correlated with the cataract density. [25] We observed relatively little differences in CST and macular volume postoperatively, perhaps relating to differences in case mix, infusion volume and the use of NSAIDS in all patients, although significant changes were observed more frequently in the HIGH group, we may speculate that this could indicate the onset of minimal macular edema.

### Intraocular pressure and RNFL changes

We found a significant rise in the IOP at day 1 in the HIGH group in the glaucoma cases, not found in the LOW group. Interestingly, Kokubun et al. in a retrospective observational study found a similar trend. [32] Thereafter there was no clear difference in the effect on IOP after 21 days, with both IOP groups showing a drop, although there was also a trend to this taking longer in the high IOP group that could be explored in future studies. We found an increase in peripapillary RNFL thickness in both groups following surgery with no difference between groups including in the glaucoma groups. Longer term follow-up with perimetry pre and postoperatively is needed to investigate the effect of infusion pressure more completely.

### Limitations

We accept that there are several limitations to our study. Importantly, it was designed as a feasibility study with range of exploratory endpoints to guide future studies. A sample size to test for equivalence of CDE between the two groups was chosen based on a conservative estimates from a previous study carried out [13]. It was not powered to assess all the endpoints examined and may have missed detection of small differences between the two groups for other endpoints. Three surgeons carried out all the surgeries and inter-surgeon variability could have masked differences. Longer follow-up, measurements of intraocular inflammation and visual fields would have added to the interpretation of our findings.

## Conclusion

In conclusion, using low IOP with standardized aspiration fluidic settings on the Centurion active sentry system did not decrease surgical efficiency or increase complication rates compared to higher pressures. It is important to note however that we excluded eyes with dense cataracts and other complex scenarios. Low IOP phacoemulsification caused fewer short-term changes in the retinal microvasculature than higher pressure, the long-term significance of which is unknown. We advocate the need of further research combining larger sample sizes, longer follow up and objective measures of inflammation to enhance our understanding on low IOP settings during cataract surgery.

## Supplementary Information

Below is the link to the electronic supplementary material.

ESM 1 (DOCX 38.0 KB)
