# Supplementary material for: The effect of intraocular pressure during phacoemulsification in patients with either diabetic retinopathy or glaucoma; a randomized controlled feasibility trial
Source: Graefes Arch Clin Exp Ophthalmol. 2025 Apr 29;263(8):2277–88. doi: 10.1007/s00417-025-06839-0 (PMC12414084; doi:10.1007/s00417-025-06839-0)
Supplement: Supplementary file 1 — (DOCX 38.0 KB) [file 417_2025_6839_MOESM1_ESM.docx]

Supplementary table 1: Surgical parameter presets during study

**LOW group**

| Surgical mode | Intraocular pressure | Vacuum | Aspiration flow |
| --- | --- | --- | --- |
| Sculpt | 30mmHg | 100 | 20 |
| Quadrant removal | 30mmHg | 600 | 40 |
| Epinucleus | 30mmHg | 600 | 40 |
| Cortex | 30mmHg | 650 | 40 |
| OVD removal | 30mmHg | 700 | 50 |

**HIGH group**

| Surgical mode | Intraocular pressure | Vacuum | Aspiration flow |
| --- | --- | --- | --- |
| Sculpt | 60mmHg | 100 | 20 |
| Quadrant removal | 60mmHg | 600 | 40 |
| Epinucleus | 60mmHg | 600 | 40 |
| Cortex | 60mmHg | 650 | 40 |
| OVD removal | 60mmHg | 700 | 50 |

Supplementary table 2: Baseline variables of patients with glaucoma

| Characteristic | HIGH [N=16] | LOW [N=13] | P-value |
| --- | --- | --- | --- |
|  |  |  |  |
| Baseline visual acuity (ETDRS letters) | 64 [56, 70] {20, 77} | 57 [52, 63] {5, 75} | 0.19 |
| Baseline IOP (mmHg) | 15.2 ± 3.4 {11, 21} | 16.5 ± 3.4 {12, 24} | 0.30 |
| Axial length (mm) | 24.0 ± 1.9  {20.1, 25.9} | 23.3 ± 1.5  {20.0, 25.4} | 0.29 |
| Central corneal thickness (microns) | 526 ± 42 {461, 623} | 515 ± 33 {466, 564} | 0.49 |
| Anterior chamber depth (mm) | 3.3 ± 0.3 {3.0, 3.8} | 3.1 ± 0.5 {2.2, 4.0} | 0.17 |
| Pupil size – Small  – Medium  – Large | 3 (19%)  8 (50%)  5 (31%) | 2 (15%)  6 (46%)  5 (38%) | 0.92 |
| Mean global RNFL | 70 ± 19 {34, 104} | 65 ± 14 {45, 97} | 0.40 |
| Number categorised as being outside normal limits for global RNFL. | 11 (69%) | 9 (69%) | 0.98 |
| Subfoveal choroidal thickness (microns) | 173 ±69 | 191±37 | 0.41 |
| Hyper-reflective foci present | 2 (13%) | 2 (17%) | 0.76 |

Summary statistics are number (percentage), mean ± standard deviation {range}, or median [inter-quartile range] {range}

Supplementary table 3: Baseline variables of patients with diabetic retinopathy.

| Characteristic | HIGH [N=21] | LOW [N=20] | P-value |
| --- | --- | --- | --- |
|  |  |  |  |
| Background retinopathy  Pre-proliferative retinopathy  Proliferative retinopathy | 7 (33%)  5 (24%)  9 (43%) | 7 (37%)  5 (26%)  7 (37%) | 0.93 |
| Maculopathy | 15 (71%) | 12 (60%) | 0.44 |
| Previous laser | 15 (71%) | 12 (60%) | 0.44 |
| Previous vitrectomy | 2 (10%) | 0 (0%) | 0.49 |
| Previous anti VEGFs | 8 (38%) | 4 (20%) | 0.20 |
| Baseline Va (ETDRS letters) | 62 [50, 68] {0, 79} | 64 [55, 72] {3, 80} | 0.40 |
| Baseline IOP (mmHg) | 15.0 ± 3.0 {10, 20} | 16.6 ± 3.6 {12, 24} | 0.14 |
| Axial length (mm) | 23.7 ± 1.2  {22.5, 25.7} | 23.5 ± 1.0  {21.7, 25.3} | 0.68 |
| CCT (microns) | 550 ± 28 {490, 601} | 558 ± 39 {488, 632} | 0.44 |
| ACD (mm) | 2.9 ± 0.4 {2.1, 3.6} | 2.9 ± 0.5 {2.2, 4.3} | 0.90 |
| Pupil size – Small  – Medium  – Large | 3 (15%)  11 (55%)  6 (30%) | 4 (20%)  11 (55%)  5 (25%) | 0.89 |
| Baseline CST | 280 [258, 325]  {175, 999} | 269 [249, 297]  {223, 999} | 0.35 |
| Macular volume | 8.4 ± 0.7 {7.1, 10.3} | 8.5 ± 0.7 {6.8, 10.0} | 0.92 |
| Subfoveal choroidal thickness (microns) | 200 ±49 | 227±61 | 0.13 |
| Hyper-reflective foci present | 13 (65%) | 7 (44%) | 0.20 |

Summary statistics are number (percentage), mean ± standard deviation {range}, or median [inter-quartile range] {range}. The presence of maculopathy was based on the UK diabetic eye screening program definition [22].

Supplementary table 4: Variables in patients with glaucoma

| Outcome | Timepoint | HIGH group (n=37) | | LOW group (n=33) | | Difference between group |
| --- | --- | --- | --- | --- | --- | --- |
|  |  | Mean ± SD | P-value | Mean ± SD | P-value | P-value |
| Visual acuity, ETDRS letters | Baseline    Day 1  Day 21  Day 40 | 60 ± 15  65 ± 18  73 ± 10  76 ± 16 | 0.16  **<0.001**  **<0.001** | 52 ± 22  59 ± 20  65 ± 19  72 ± 14 | 0.14  **0.005**  **<0.001** | 0.91  0.37  0.92 |
| Intraocular pressure, mmHg | Baseline    Day 1  Day 21  Day 40 | 15.2 ± 3.4  22.8 ± 10.6  16.9 ± 4.8  13.3 ± 3.3 | **0.004**  0.23  0.08 | 16.5 ± 3.4  19.0 ± 8.1  15.4 ± 3.7  14.9 ± 4.3 | 0.29  0.23  0.17 | 0.10  0.21  0.47 |
| Central corneal thickness, microns | Baseline    Day 1  Day 21  Day 40 | 526 ± 42  588 ± 100  530 ± 43  520 ± 45 | **0.005**  0.44  0.09 | 515 ± 33  605 ± 84  515 ± 34  516 ± 32 | **0.001**  0.91  0.91 | 0.28  0.41  0.27 |
| Anterior chamber depth, mm | Baseline    Day 1  Day 21  Day 40 | 3.3 ± 0.3  4.8 ± 0.8  5.1 ± 0.5  5.3 ± 0.5 | **<0.001**  **<0.001**  **<0.001** | 3.1 ± 0.5  4.4 ± 0.9  5.0 ± 0.3  4.9 ± 0.4 | **<0.001**  **<0.001**  **<0.001** | 0.37  0.57  0.10 |
| Central macular thickness, Microns | Baseline    Day 1  Day 21  Day 40 | 269 ± 27  271 ± 28  275 ± 31  276 ± 29 | 0.09  **0.02**  **0.006** | 326 ± 206  327 ± 205  274 ± 38  277 ± 41 | 0.34  0.37  0.39 | 0.58  0.70  0.80 |
| Macular Volume,  mm^3^ | Baseline    Day 1  Day 21  Day 40 | 7.9 ± 0.8  7.9 ± 0.8  8.1 ± 0.8  8.1 ± 0.8 | 0.57  **<0.001**  **<0.001** | 8.0 ± 0.9  8.1 ± 0.8  8.0 ± 0.7  8.1 ± 0.7 | 0.12  0.80  0.92 | 0.27  0.35  0.52 |
| Global peripapillary retinal nerve fibre layer thickness, microns | Baseline    Day 1  Day 21  Day 40 | 70 ± 19  70 ± 19  73 ± 19  73 ± 19 | **0.04**  **0.001**  **<0.001** | 65 ± 14  67 ± 10  68 ± 16  71 ± 14 | **0.003**  **0.005**  **0.003** | 0.05  0.95  0.13 |
| Subfoveal choroidal thickness, microns | Baseline    Day 1  Day 21  Day 40 | 173 ± 69  164 ± 68  176 ± 69  177 ± 67 | **0.006**  0.60  0.37 | 191 ± 37  184 ± 40  186 ± 49  185 ± 50 | 0.13  0.43  0.34 | 0.84  0.77  0.95 |
| Retinal hyper-reflective foci, number | Baseline    Day 1  Day 21  Day 40 | 2 (13%)  1 (8%)  1 (6%)  3 (19%) | 1.00  0.32  0.32 | 2 (17%)  2 (18%)  3 (23%)  3 (23%) | 1.00  1.00  1.00 | 0.44  0.19  0.77 |
| FAZ area | Baseline    Day 21  Day 40 | 0.29 ± 0.09  0.28 ± 0.10  0.28 ± 0.14 | **0.02**  0.60 | 0.23 ± 0.11  0.27 ± 0.11  0.25 ± 0.12 | 0.20  0.71 | **0.02**  0.35 |
| FAZ perimeter | Baseline    Day 21  Day 40 | 2.20 ± 0.40  2.05 ± 0.39  2.09 ± 0.50 | **0.02**  0.25 | 1.93 ± 0.52  2.04 ± 0.52  1.92 ± 0.57 | 0.73  0.91 | 0.08  0.57 |
| Superficial capillary plexus density | Baseline    Day 21  Day 40 | 40.5 ± 3.9  43.7 ± 3.3  43.8 ± 4.9 | **<0.001**  **0.002** | 39.8 ± 5.6  41.9 ± 5.5  42.0 ± 5.0 | 0.10  0.27 | 0.27  0.74 |

Supplementary table 5: Variables in patients with diabetic retinopathy

| Outcome | Timepoint | HIGH group (n=37) | | LOW group (n=33) | | Difference between group |
| --- | --- | --- | --- | --- | --- | --- |
|  |  | Mean ± SD | P-value | Mean ± SD | P-value | P-value |
| Visual acuity, ETDRS letters | Baseline    Day 1  Day 21  Day 40 | 54 ± 23  57 ± 20  69 ± 18  74 ± 14 | 0.56  **0.001**  **<0.001** | 57 ± 20  60 ± 22  76 ± 9  77 ± 7 | 0.85  **0.007**  **0.001** | 0.78  0.20  0.52 |
| Intraocular pressure, mmHg | Baseline    Day 1  Day 21  Day 40 | 15.0 ± 3.0  17.8 ± 6.3  15.0 ± 3.4  15.0 ± 3.0 | **0.02**  0.78  1.00 | 16.6 ± 3.6  23.0 ± 7.8  15.7 ± 4.0  14.7 ± 3.1 | **<0.001**  0.38  **0.02** | 0.08  0.86  0.8 |
| Central corneal thickness, microns | Baseline    Day 1  Day 21  Day 40 | 550 ± 28  673 ± 143  561 ± 32  561 ± 34 | **<0.001**  **0.02**  **0.03** | 558 ± 39  707 ± 137  577 ± 39  564 ± 35 | **<0.001**  **0.004**  0.12 | 0.53  0.44  0.668 |
| Anterior chamber depth, mm | Baseline    Day 1  Day 21  Day 40 | 2.9 ± 0.4  4.6 ± 0.7  5.2 ± 0.5  5.1 ± 0.5 | **<0.001**  **<0.001**  **<0.001** | 2.9 ± 0.5  4.4 ± 0.8  4.9 ± 0.5  5.2 ± 0.6 | **<0.001**  **<0.001**  **<0.001** | 0.65  0.14  0.87 |
| Central macular, Microns | Baseline    Day 1  Day 21  Day 40 | 326 ± 167  315 ± 168  288 ± 51  298 ± 64 | 0.83  0.30  0.43 | 310 ± 166  272 ± 37  284 ± 41  291 ± 53 | 0.31  0.46  0.64 | 0.29  0.82  0.77 |
| Macular Volume,  mm^3^ | Baseline    Day 1  Day 21  Day 40 | 8.5 ± 0.7  8.4 ± 0.9  8.7 ± 0.8  8.8 ± 1.0 | 0.42  0.11  **0.04** | 8.5 ± 0.7  8.3 ± 0.6  8.7 ± 0.6  8.8 ± 0.7 | 0.14  0.15  **0.02** | 0.40  0.68  0.78 |
| Global peripapillary retinal nerve fibre layer thickness, microns | Baseline    Day 1  Day 21  Day 40 | 89 ± 11  89 ± 10  93 ± 13  95 ± 13 | 0.19  **0.005**  **<0.001** | 88 ± 13  90 ± 13  94 ± 13  95 ± 11 | 0.30  **<0.001**  **<0.001** | 0.74  0.40  0.50 |
| Global ONL | Baseline    Day 1  Day 21  Day 40 | 3 (14%)  3 (19%)  2 (10%)  2 (10%) | 1.00  0.32  0.32 | 3 (15%)  2 (11%)  8 (12%)  2 (10%) | 1.00  1.00  0.32 | 0.53  0.86  0.96 |
| Subfoveal choroidal thickness, microns | Baseline    Day 1  Day 21  Day 40 | 200 ± 49  201 ± 47  217 ± 46  214 ± 46 | 0.82  0.21  **0.04** | 227 ± 61  205 ± 75  228 ± 61  238 ± 58 | 0.16  0.48  0.10 | 0.39  0.85  0.79 |
| Retinal hyper-reflective foci, number | Baseline    Day 1  Day 21  Day 40 | 13 (65%)  12 (67%)  14 (70%)  15 (71%) | 0.32  0.32  0.56 | 7 (44%)  9 (50%)  9 (53%)  10 (50%) | 1.00  0.32  0.32 | 0.31  0.29  0.16 |
| FAZ area | Baseline    Day 21  Day 40 | 0.29 ± 0.15  0.29 ± 0.14  0.28 ± 0.16 | 0.34  0.52 | 0.32 ± 0.12  0.32 ± 0.12  0.33 ± 0.12 | 0.96  0.65 | 0.51  0.96 |
| FAZ perimeter | Baseline    Day 21  Day 40 | 2.20 ± 0.81  2.16 ± 0.73  2.20 ± 0.88 | 0.27  0.81 | 2.26 ± 0.49  2.25 ± 0.43  2.33 ± 0.50 | 0.37  0.81 | 0.18  0.96 |
| Superficial capillary plexus density | Baseline    Day 21  Day 40 | 38.0 ± 4.2  40.9 ± 5.7  40.4 ± 6.6 | 0.08  0.16 | 40.4 ± 6.6  38.7 ± 5.8  39.0 ± 6.3 | 0.39  0.59 | 0.14  0.28 |
